# PFKL Inhibition by DT‐13: A Novel Approach to Combat Hepatocellular Carcinoma

**DOI:** 10.1155/ijh/5211859

**Published:** 2025-12-12

**Authors:** Qiang Yu, Liangning Hu, Chenfei Tan, Min Gao, Zhenzhen Wen

**Affiliations:** ^1^ Department of Gastroenterology, Sir Run Run Shaw Hospital, Zhejiang University School of Medicine, Hangzhou, Zhejiang, China, zju.edu.cn; ^2^ 2nd Department of Hematology, Sir Run Run Shaw Hospital, Zhejiang University School of Medicine, Hangzhou, Zhejiang, China, zju.edu.cn

**Keywords:** aerobic glycolysis, hepatocellular carcinoma, *Liriope muscari* Baily Saponin C, Phosphofructokinase-1 liver type, sorafenib

## Abstract

Aerobic glycolysis modulates proliferation, apoptosis, immune evasion, and targeted drug resistance in hepatocellular carcinoma (HCC) patients. Therefore, inhibiting aerobic glycolysis could represent a novel chemotherapeutic strategy for HCC. The effects of *Liriope muscari* Baily′s Saponin C (DT‐13), a novel compound isolated from the traditional Chinese medicine *Liriope muscari* (Decne) Baily, on HCC and its underlying mechanism remain unknown. This study revealed that DT‐13 induces apoptosis and inhibits the in vivo and in vitro proliferation of HCC cells. Furthermore, DT‐13 significantly reduced glucose consumption and lactate production. Moreover, it was observed that DT‐13 could inhibit Phosphofructokinase‐1 liver (PFKL) type via c‐myc signaling to modulate the aerobic glycolysis, proliferation, and apoptosis of HCC. Moreover, DT‐13 improved the anticancer effects of sorafenib in HCC. In summary, this study provided evidence for the potential application of DT‐13 in HCC treatment.

## 1. Introduction

Liver cancer has become a severe health burden with an estimated 600,000 to 900,000 annual deaths globally. Hepatocellular carcinoma (HCC) represents the main pathological type of primary liver cancer [1]. The FDA has recommended sorafenib, an oral multikinase inhibitor, as the first‐line therapy for advanced HCC, but due to its lack of significant efficacy [2, 3], more effective drugs for HCC treatment are urgently required [4].

In recent years, cancer cell metabolism has become a research hotspot. Otto Warburg reported that, even in aerobic conditions, rat HCC cells were significantly dependent on glycolysis rather than oxidative phosphorylation (OXPHOS), a phenomenon termed “aerobic glycolysis” or the “Warburg effect” [5]. Furthermore, increased aerobic glycolysis has been associated with various human carcinomas, including HCC [6], gastric cancer [7], and colorectal cancer [8]. It has been observed that aerobic glycolysis produces ATP and other essential biosynthetic intermediates, which facilitate proliferation. Lactate produced by aerobic glycolysis acidifies the tumor microenvironment [9], promoting tumor angiogenesis and metastasis while inhibiting the antitumor activity of immune cells within this microenvironment. In cancer glycolysis, three enzymes have been identified as the rate‐limiting enzymes, including Pyruvate Kinase Type M2 (PKM2), Phosphofructokinase‐1 (PFK1), and Hexokinase 2 (HK2). PFK1 utilizes ATP for catalyzing the conversion of fructose 6‐phosphate (F‐6‐P) to fructose 1,6‐bisphosphate (F‐1,6‐BP). Furthermore, PFK1 has three isoforms in mammals: the liver type (PFKL), the muscle type (PFKM), and the plasma type (PFKP). In HCC, PFKL is the primary expression isoform of PFK1 [10], which promotes aerobic glycolysis and cancer cell proliferation, while inhibiting apoptosis [11, 12]. Li et al. reported that epigallocatechin‐3‐gallate (EGCG)–induced PFKL inhibition suppressed HCC cell proliferation and induced apoptosis. Moreover, EGCG improved HCC′s sensitivity to sorafenib [13]. Therefore, based on these data, PFKL could potentially be targeted for the treatment of HCC.

*Liriope muscari* Baily Saponin C (DT‐13), a saponin monomer of the dwarf lilyturf tuber, is isolated from traditional Chinese medicine *Liriope muscari* (Decne) Baily (Chinese name: Duanting Shan‐maidong). DT‐13 could exert anticancer effects on a variety of cancers, including gastric cancer [14], colorectal cancer [15], and acute myeloid leukemia [16]. Furthermore, Wei et al. [15] indicated that DT‐13 inhibits aerobic glycolysis in colorectal cancer by downregulating GLUT1, in vivo and in vitro. Moreover, Yu et al. [14] revealed that DT‐13 could be synergistically combined with topotecan to inhibit glycolysis in gastric cancer. It has been observed that DT‐13 exerts its anticancer effects by modulating other important cancer phenotypes, such as autophagy [17], differentiation [16], and mitotic arrest [18]. However, DT‐13′s effects on HCC, as well as its glycolysis metabolism, remain undetermined.

This study revealed that DT‐13 triggered apoptosis in HCC cells and inhibited their proliferation and aerobic glycolysis by the c‐myc/PFKL pathway. Moreover, DT‐13 improved HCC′s in vitro and in vivo sensitivity of cells to sorafenib. DT‐13 indicated good safety profiles in both in vivo and in vitro experiments. These findings provide the basis for identifying reliable and effective therapeutic methods for HCC.

## 2. Materials and Methods

### 2.1. Reagents

DT‐13 (over 98% pure) and sorafenib were purchased from Yuanye Bio‐Technology Co. (Shanghai, China). Reagents for measuring the level of ALT, creatinine, glucose, lactate, and ATP were obtained from Jiancheng Bioengineering Institute (Nanjing, China). DMEM and FBS were purchased from Gibco (Thermo Fisher Scientific, Waltham, United States). Reagents for measuring the level of NADPH/NADP^+^ were obtained from Beyotime Biotech Inc. (Shanghai, China).

### 2.2. Cell Culture

The normal human liver cell line THLE‐2 (official cell line name: THLE‐2, RRID: CVCL_3803) and human HCC cell lines Huh7 (official cell line name: Huh‐7, RRID: CVCL_0336), Hep3B (official cell line name: Hep 3B2.1‐7, RRID: CVCL_0326), and LM3 (official cell line name: HCC‐LM3, RRID: CVCL_6832) were purchased from the Chinese Academy of Sciences Cell Bank (Shanghai, China). Cultures of the HCC cell lines and THLE‐2 were then established in DMEM containing 100 g/mL streptomycin, 100 U/mL penicillin, and 10% FBS prior to incubation at 37°C in a 5% CO_2_ humidified incubator. The sorafenib‐resistant Huh7 (Huh7‐SR) cell line was cultured following standard protocol [19]. Huh7‐SR cell was cultured in DMEM containing 1 *μ*M sorafenib, which was replaced with fresh DMEM without sorafenib 24 h before analysis.

### 2.3. CCK‐8 Assay

A cell suspension of concentration 1 × 10^4^/mL was added to each well of a 96‐well plate, and following a 24‐h incubation, the medium was removed before adding a fresh one containing varying concentrations of DT‐13 and/or sorafenib. After 48 h, 10 *μ*L CCK‐8 solution (Yeasen, Shanghai, China) was added to each well. This was followed by a 2‐h incubation at 37°C, with absorbance readings eventually recorded at 450 nm using a microplate reader.

### 2.4. Colony Formation Assay

To each well of a six‐well plate, 1000 cells were added, and after a 24‐h incubation, the medium was replaced with fresh medium containing DT‐13 and/or sorafenib. This was followed by a 14‐day incubation, with the cells subsequently fixed using 0.1% crystal violet (Servicebio, Wuhan, China).

### 2.5. Flow Cytometry

Cells were incubated for 24 h in a six‐well plate. Then, the medium was replaced and supplemented with DT‐13 or sorafenib. After incubating for another 48 h, cells were collected and fixed with Annexin V/propidium iodide (Beyotime Biotechnology, Shanghai, China) and detected by flow cytometry.

### 2.6. Biochemical Assays

Lactate levels in both tumor tissues and culture media were measured with a specific lactate assay kit. The levels of ALT and creatinine in serum samples were assessed utilizing the recommended biochemical assay kits, following the manufacturer′s guidelines. The levels of ATP and NADPH in HCC cells were measured with the relative biochemical assay kits. The glucose uptake by cells was quantified as described in earlier studies, with the data adjusted relative to the protein content [20]. pH of cell supernatant was measured using LAQUA F‐73 Benchtop pH/ORP/Ion/Temperature Meter (HORIBA Advanced Techno, Tokyo, Japan).

### 2.7. Western Blotting

Cells or tumor tissues were incubated in RIPA lysis buffer containing protease inhibitors in order to extract total protein, whose concentration was subsequently determined with a BCA kit (Beyotime Biotechnology, Shanghai, China). The extracted protein (30 *μ*g) was then separated by electrophoresis before being transferred to a cellulose acetate membrane (Servicebio, Wuhan, China). After blocking with 5% BSA, the membranes were incubated with the primary antibodies, with both the blocking and incubation steps performed overnight at 4°C. PBST was then used to wash the membranes prior to a 1‐h incubation at room temperature with appropriate secondary antibodies. A second washing step was performed using PBST, with membranes finally scanned by Amersham Imager 680 RGB (General Electric Company, Boston, United States). Details of the primary antibodies used are provided in Table S1.

### 2.8. Reverse‐Transcriptase (RT)‐PCR

TRIzol reagent (Servicebio, Wuhan, China) was used to extract total RNA from cells prior to reverse transcription with a reverse transcription kit (Takara, Dalian, China). The resulting cDNA was then amplified by RT‐PCR using an RT‐PCR kit (Yeasen, Shanghai, China), with information regarding the selected primers provided in Table S2. The 2^−*Δ* *Δ*Ct^ method was eventually used to assess the level of gene expression.

### 2.9. Plasmid Construction, Lentivirus Packaging, and Infection

Lentiviruses for PFKL overexpression (PFKL‐OE) or c‐myc overexpression (c‐myc‐OE) and knockdown were synthesized by General Biol (Anhui, China). A recombinant plasmid for PFKL‐OE or c‐myc‐OE was also constructed by amplifying and subsequently cloning the full‐length cDNA encoding the PFKL sequence into the VP018‐U6‐MCS‐PGK‐PURO vector. Moreover, a recombinant plasmid expressing PFKL‐shRNA (sh‐PFKL) or c‐myc‐shRNA (sh‐c‐myc) was generated by cloning double‐stranded oligonucleotides into the PLVX‐PURO vector. For the negative control, an empty vector (PFKL empty vector [PFKL‐EV] or c‐myc empty vector [c‐myc‐EV]) was used.

HCC cells at a density of 3 × 10^4^/L were incubated for 24 h in a 12‐well plate before replacing the culture medium with fresh one containing 5 *μ*g/mL polybrene and lentivirus. After another 24‐h incubation, the cells were treated with puromycin for 48 h to select positive cells. RT‐PCR and western blotting were eventually performed to assess infection efficiency.

### 2.10. In Vivo Experiments

Five‐week‐old male BALB/C nude mice, obtained from Zhejiang University School of Medicine (Hangzhou, China), were kept in a standard animal laboratory where they could freely access water and food. The animals were then injected with LM3 cell suspensions (5 × 10^6^) in PBS into the upper lateral area, and once the tumors reached detectable sizes, 12 mice were assigned to one of the following two groups: one receiving daily 1% DMSO for 16 days and the other receiving 2.5 mg/kg DT‐13 daily for the same period, with weight and tumor volume checks every 4 days. In a separate sorafenib combination study, 16 mice were divided into four groups: control with DMSO, DT‐13 alone, sorafenib alone, and a combination of both at daily doses of 2.5 mg/kg for 16 days. At the end of the 16 days of treatment, the mice were anaesthetized with 40 mg/kg 1.25% pentobarbital by intraperitoneal injection and sacrificed by cervical dislocation. The tumors, lungs, hearts, kidneys, and blood were obtained for further experiments. This study adheres to the ARRIVE guidelines.

### 2.11. Hematoxylin and Eosin (H&E) Staining, Immunohistochemical (IHC) Staining, and TUNEL Assay

After fixing the tumor, liver, lung, heart, and kidney tissues in paraformaldehyde, the samples were paraffinized before being cut into 3‐*μ*m sections. This was followed by H&E staining for histology. The tissues were then processed for IHC with Ki67 primary and secondary antibodies after dewaxing, dehydrating, antigen retrieval, and blocking. TUNEL assays were conducted on tumor sections following similar preparation steps and proteinase digestion and then incubated in the TUNEL mix. Images from these procedures were captured using a microscope.

### 2.12. Statistical Analysis

Experiments in the current study were repeated at least three times before using Student′s *t*‐tests for pairwise comparisons of groups. In this case, statistically significant differences were indicated by *p* values of less than 0.05.

## 3. Results

### 3.1. DT‐13 Inhibited the Proliferation and Induced Apoptosis of HCC Cells

DT‐13′s toxicity on normal liver (THLE‐2) and HCC cell lines was investigated using CCK‐8. Overall, DT‐13 suppressed the proliferation of all HCC cell lines but had no effect on the viability of the THLE‐2 (Figure 1a). The half maximal inhibitory concentration (IC_50_) of DT‐13 on Huh7, Hep3B, and HCC‐LM3 cells at 48 h was 18.57, 17.60, and 14.02 *μ*M, respectively, with the latter two cell lines selected for subsequent analyses. The colony formation assay revealed that DT‐13 inhibited HCC cell proliferation and colony formation ability (Figure 1b). Moreover, it also inhibited the protein expression of PCNA, validating that DT‐13 can reduce cell proliferation in vitro (Figure 1d). Furthermore, flow cytometry analysis revealed that concentrations of 5, 10, and 20 *μ*M DT‐13 increased the apoptotic rate of HCC‐LM3 cells, whereas the apoptotic rate of Hep3B cells was increased at 10 and 20 *μ*M concentrations (Figure 1c). Bcl‐2 functions as a protein that inhibits apoptosis, and western blot analysis indicated that its expression in HCC cell lines was reduced by DT‐13 (Figure 1d). These results suggest that in vitro treatment of HCC cells with DT‐13 induced apoptosis.

Figure 1Results of in vitro and in vivo experiments showing DT‐13′s effects on HCC cell proliferation and apoptosis. (a) Cytotoxicity of DT‐13 on normal liver and HCC cell lines as detected by CCK‐8 assay. (b) DT‐13′s effects on colony formation of HCC cell lines. (c) Flow cytometry analysis of apoptosis rate following 48 h of DT‐13 treatment (*n* = 3,  ^∗^ *p* < 0.05 vs. DT‐13 [0 *μ*M]). (d) PCNA and Bcl‐2 expression as detected by western blot. (e) Body weight changes of mice after DT‐13 treatment (*n* = 6, *p* > 0.05). (f) Tumor volume changes of mice after DT‐13 treatment (*n* = 6,  ^∗^ *p* < 0.05 vs. vehicle). (g) Ki67 staining and TUNEL staining of tumor tissues (magnification 200×). (h) Expression of PCNA and Bcl‐2 proteins in tumors.

**Figure:**
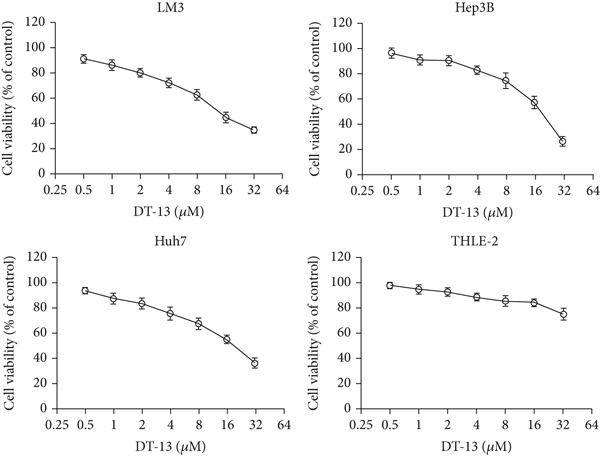
(a)

**Figure:**
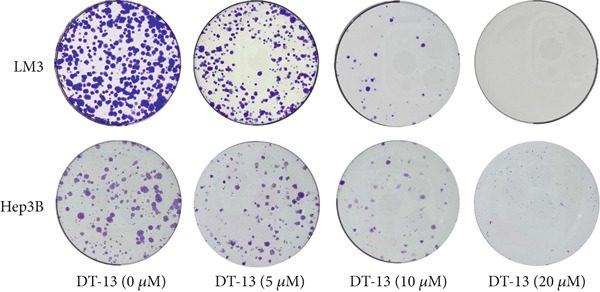
(b)

**Figure:**
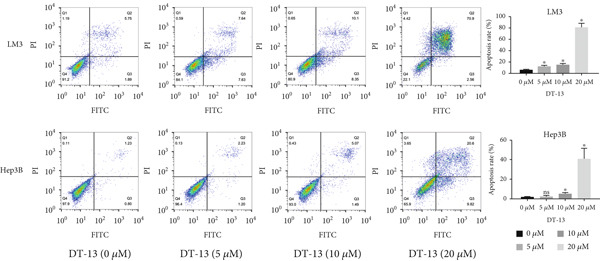
(c)

**Figure:**
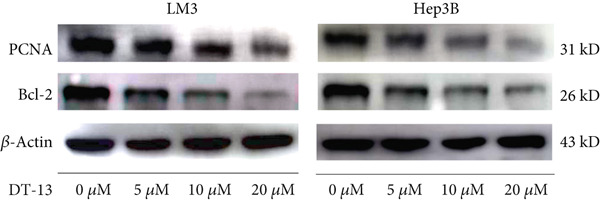
(d)

**Figure:**
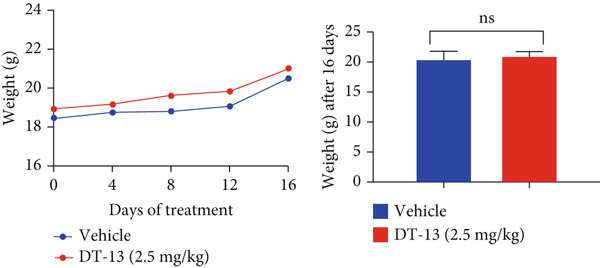
(e)

**Figure:**
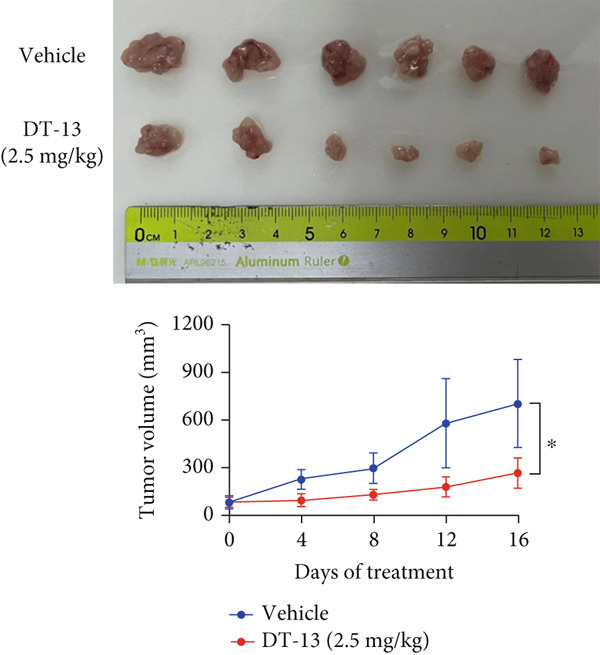
(f)

**Figure:**
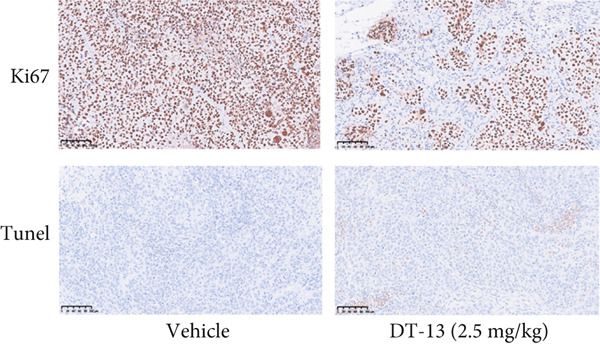
(g)

**Figure:**
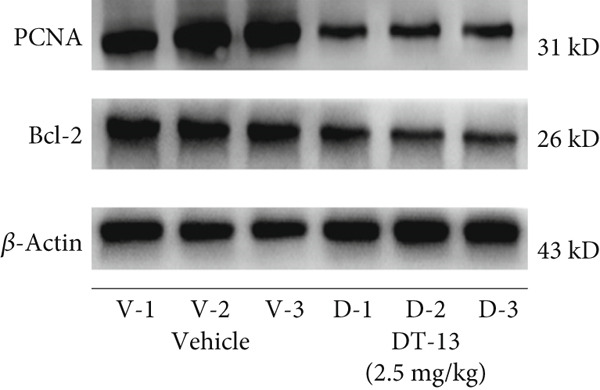
(h)

This study also established a xenograft model to evaluate the effects of DT‐13 on HCC in vivo. As shown in Figure 1e, the 16‐day oral administration of 2.5 mg/kg DT‐13 had no effect on the body weight of mice; however, it significantly decreased the overall size of the tumors (Figure 1f). The TUNEL assay, as well as IHC analysis, further revealed that the DT‐13 group had a higher number of apoptotic cells and a reduced number of Ki‐67‐positive cells, respectively, compared with the vehicle group (Figure 1g). Similarly, western blot analysis of tumor tissues confirmed that DT‐13 suppressed PCNA and Bcl‐2 expression (Figure 1h).

Overall, the findings of both in vitro and in vivo experiments showed that DT‐13 inhibited the proliferation of HCC cells while inducing apoptosis.

### 3.2. DT‐13 Is Safe In Vivo and In Vitro

No pathological changes were noted in the animals′ major organs after DT‐13 treatment (Figure 2a). Furthermore, DT‐13 had no effect on the levels of ALT and creatinine (Figure 2b). Moreover, flow cytometry indicated that DT‐13 did not alter the apoptosis rate of THLE‐2 cells (Figure 2c). These data indicate the in vitro and in vivo safety of DT‐13 treatment, consistent with the results of previous studies [21, 22].

Figure 2DT‐13′s toxicity profile through in vitro and in vivo experiments. (a) H&E staining of kidney, lung, liver, and heart tissues from mice (magnification 200×). (b) Serum levels of creatinine and ALT (*n* = 6, *p* > 0.05). (c) Apoptosis rate of THLE‐2 cells after DT‐13 treatment (*n* = 3, *p* > 0.05). (d) Lactate production and glucose uptake of THLE‐2 cells after DT‐13 treatment (*n* = 3, *p* > 0.05).

**Figure:**
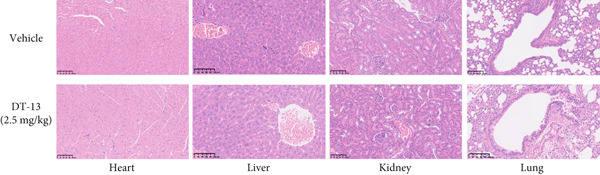
(a)

**Figure:**
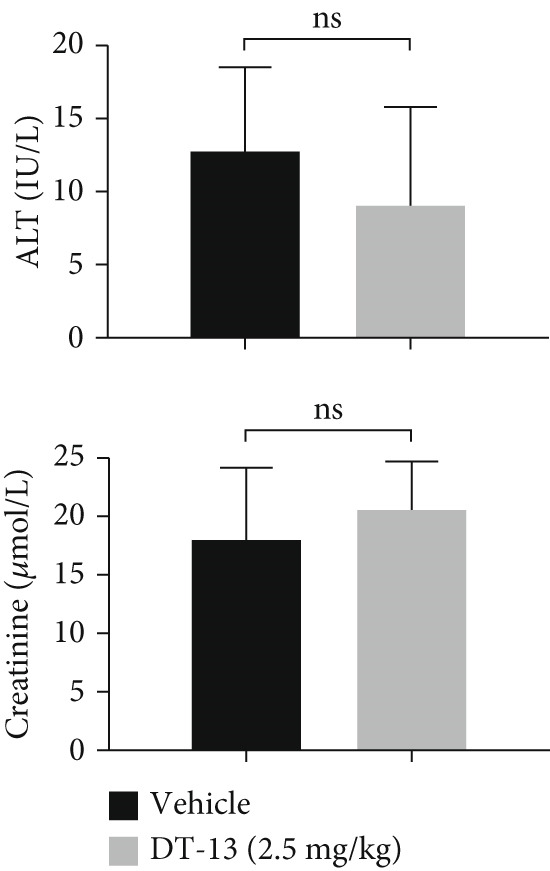
(b)

**Figure:**
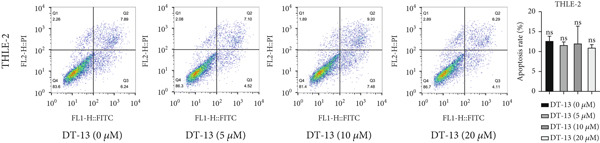
(c)

**Figure:**
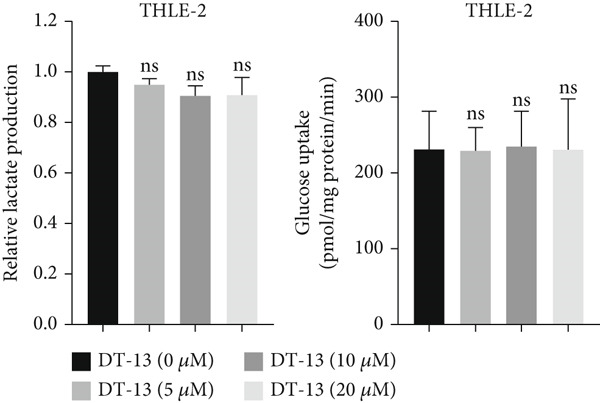
(d)

### 3.3. DT‐13 Reduced the Aerobic Glycolysis Level of HCC Cells

Glucose uptake by HCC‐LM3 and Hep3B cells and subsequent lactate production were hindered by the 48‐h treatment with DT‐13 in a dose‐dependent manner (Figure 3a). Furthermore, the oral uptake of 2.5 mg/kg DT‐13 reduced the lactate levels in tumor tissues (Figure 3d). However, in the case of THLE‐2 cells, DT‐13 had no effect on glycolysis (Figure 2d). Consistent with reduced lactate output, the pH of the culture supernatant increased after DT‐13 treatment (Figure S1A). Moreover, DT‐13 significantly downregulated ATP production in a dose‐dependent manner (Figure S1B), aligning with previous findings [15]. DT‐13 treatment also reduced NADPH levels in HCC cells (Figure S1C).

Figure 3Results of in vitro and in vivo experiments showing the suppression of aerobic glycolysis in HCC cells after DT‐13 treatment. (a) Lactate production and glucose uptake by Hep3B and LM3 cells following 48 h of treatment with DT‐13 (*n* = 3,  ^∗^ *p* < 0.05 vs. DT‐13 [0 *μ*M]). (b) mRNA expressions of seven genes related to aerobic glycolysis in HCC cells (*n* = 3,  ^∗^ *p* < 0.05 vs. DT‐13 [0 *μ*M]). (c) Protein expression of PFKL, ATP5A1, and UQCRC2 detected by western blot. (d) Lactate levels in the tumor tissues (*n* = 6,  ^∗^ *p* < 0.05 vs. vehicle). (e) Protein expression of PFKL in the tumor tissues of mice detected by western blot.

**Figure:**
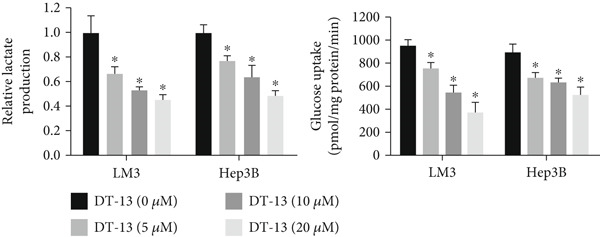
(a)

**Figure:**
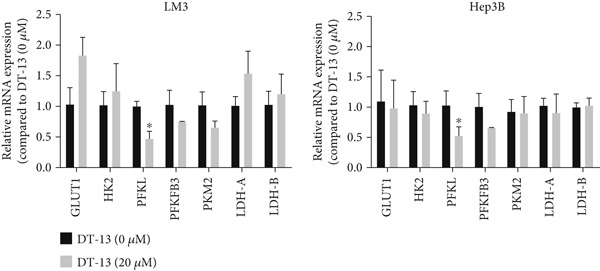
(b)

**Figure:**
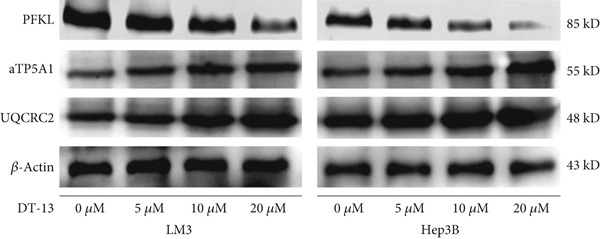
(c)

**Figure:**
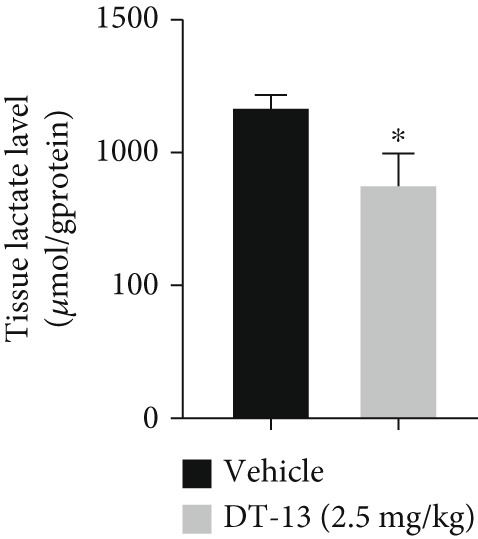
(d)

**Figure:**
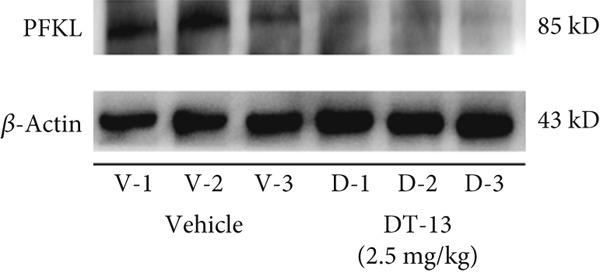
(e)

This study also assessed the potential target of DT‐13 during the inhibition of aerobic glycolysis in HCC. For this purpose, RT‐PCR analysis of HCC‐LM3 and Hep3B cells was conducted to determine the mRNA levels of seven aerobic glycolysis–related genes. It was found that, in both cell lines, the 48‐h treatment with DT‐13 significantly inhibited PFKL expression (Figure 3b). Furthermore, western blot analysis indicated that treatment with DT‐13 decreased PFKL protein levels in both animal models and cell cultures (Figure 3c,e). Moreover, to confirm the interaction between DT‐13 and PFKL, a molecular docking study was conducted to investigate the binding of DT‐13 to PFKL. Multiple docking complexes were formed, and their affinities were evaluated from the calculated binding energies. Molecular docking analysis indicated that DT‐13 exhibited strong predicted binding affinity for PFKL (Gscore = −9), interacting within a surface cavity on the surface of the PFKL protein through four hydrogen bonds (Figure S2A,B).

To determine isoform specificity, the effects of DT‐13 treatment on the expression of other phosphofructokinase family members were investigated. RT‐PCR results showed that while DT‐13 downregulated the expression of PFKP and PFKL, the changes were not statistically significant (Figure S3A). Western blot analysis indicated that DT‐13 reduced the protein expression of PFKL, while it did not affect the expression of PFKP and PFKM (Figure S3B). ATP5A1 and UQCRC2 are essential proteins in the OXPHOS process. To assess how DT‐13 modulates OXPHOS in HCC cells, the level of ATP5A1 and UQCRC2 proteins was detected. The findings suggested that HCC cells could exhibit higher OXPHOS expression following treatment with DT‐13 (Figure 3c).

Altogether, the results of both in vitro and in vivo experiments confirmed that treating HCC cells with DT‐13 inhibited aerobic glycolysis.

### 3.4. DT‐13 Modulates Aerobic Glycolysis as Well as HCC Cells′ Proliferation and Apoptosis by Inhibiting PFKL

Among the seven glycolysis‐related genes investigated, DT‐13 decreased both the mRNA and protein expression of PFKL in HCC‐LM3 and Hep3B cells, as revealed by in vivo and in vitro experiments. In cancer cells, PFKL is known to modulate aerobic glycolysis, proliferation, and apoptosis [9, 12]. Therefore, this study evaluated whether DT‐13′s inhibitory effects on PFKL modulate these processes in HCC cells.

After infecting HCC‐LM3 and Hep3B cells with a PFKL‐EV or lentiviruses containing PFKL‐OE or sh‐PFKL, the transfection efficiency was determined using RT‐PCR (Figure 4a) and western blotting (Figure 4b). The data indicated that DT‐13 inhibited aerobic glycolysis of PFKL‐OE and PFKL‐EV HCC cells. However, in sh‐PFKL HCC cells, DT‐13 did not affect lactate output and glucose consumption (Figure 4c). The results of CCK‐8 analyses and western blotting indicated that DT‐13 suppressed the cell viability and protein expression of PCNA and Bcl‐2 in PFKL‐OE and PFKL‐EV HCC cells, but not in sh‐PFKL HCC cells (Figure 4d,e).

Figure 4DT‐13 modulated HCC cells′ glycolytic activity, proliferation, and apoptosis by targeting PFKL. (a, b) Transfection was validated using RT‐PCR and western blot. (c) Effects of 20 *μ*M DT‐13 on the aerobic glycolysis of PFKL‐OE, sh‐PFKL, or PFKL‐EV HCC cells (*n* = 3,  ^∗^ *p* < 0.05 vs. PFKL‐OE, ^#^ *p* < 0.05 vs. PFKL‐EV). (d) Cell viability was measured after 48 h of DT‐13 treatment (*n* = 3,  ^∗^ *p* < 0.05 vs. PFKL‐OE, ^#^ *p* < 0.05 vs. PFKL‐EV). (e) PCNA and Bcl‐2 expression in PFKL‐OE, sh‐PFKL, or PFKL‐EV HCC cells.

**Figure:**
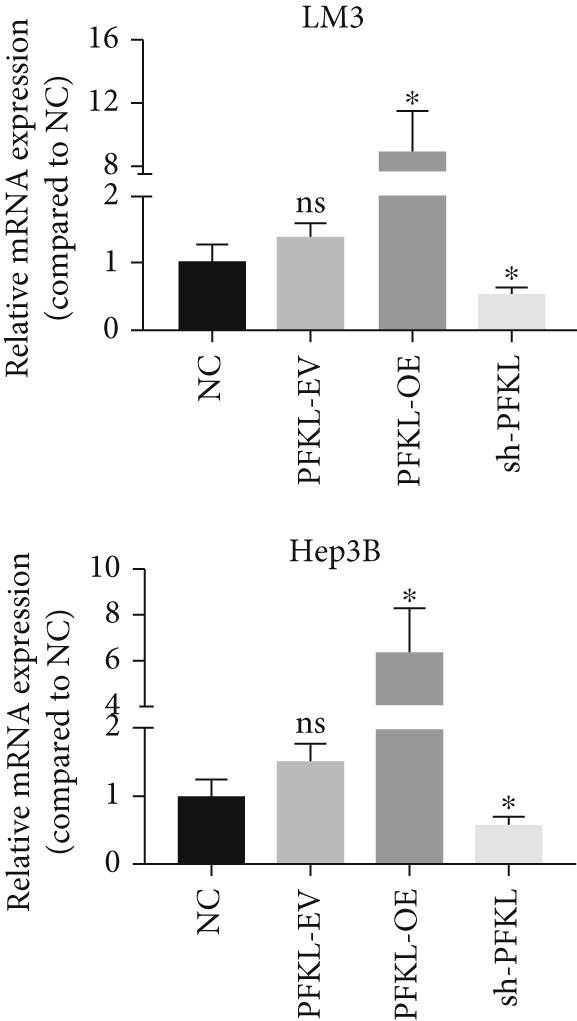
(a)

**Figure:**
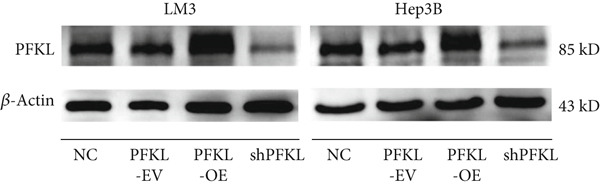
(b)

**Figure:**
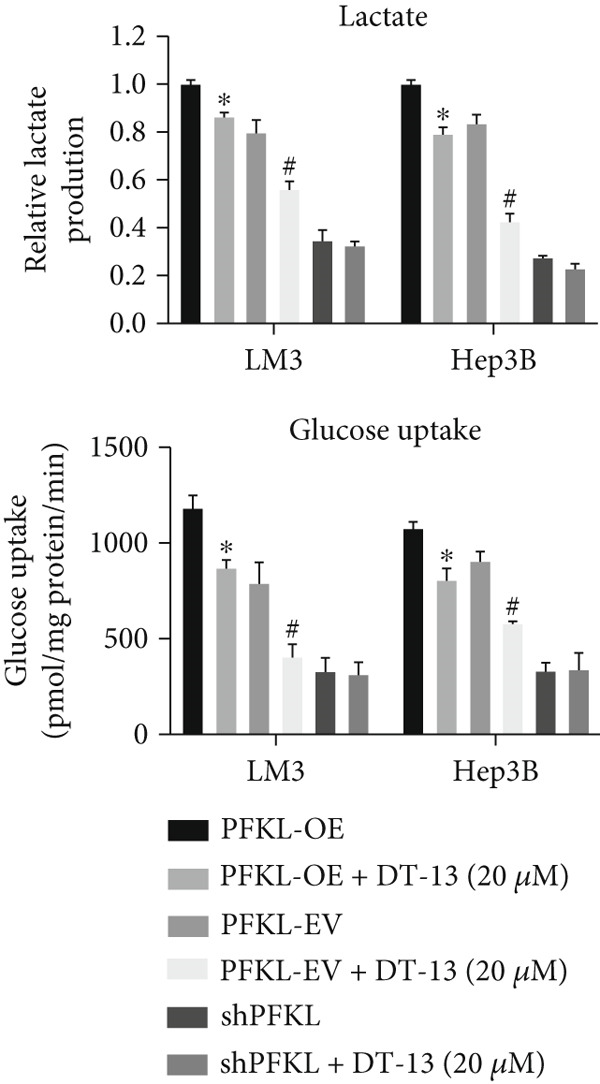
(c)

**Figure:**
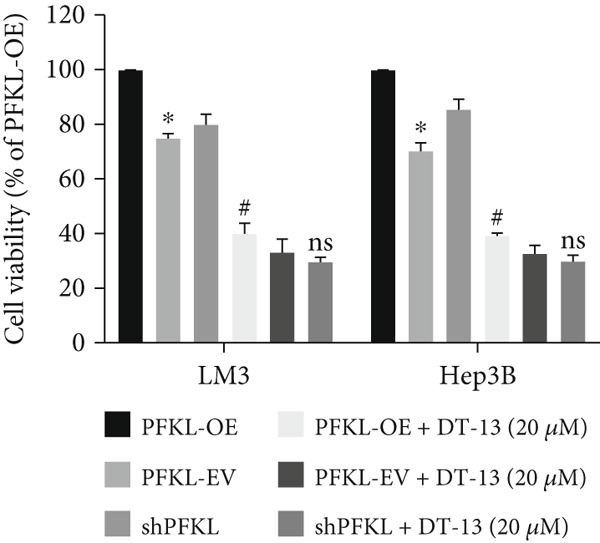
(d)

**Figure:**
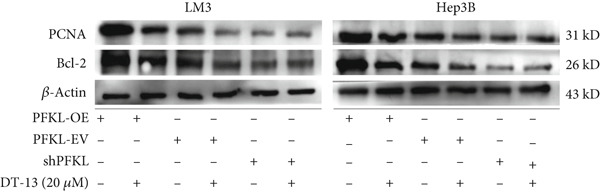
(e)

Altogether, the findings suggested that DT‐13 regulates aerobic glycolysis as well as HCC cells′ proliferation and apoptosis by targeting PFKL.

### 3.5. DT‐13 Downregulates PFKL by the c‐myc Pathway

Next, the mechanism by which DT‐13 regulates the expression of PFKL in HCC cells was further explored. Previous studies have shown that PFKL expression is regulated by multiple signaling pathways [9]. In this study, PCR analysis revealed that DT‐13 significantly suppressed c‐myc mRNA levels in both HCC cell lines (Figure 5a). Accordingly, DT‐13 reduced c‐myc protein expression in both cell culture and animal models (Figure 5b,c).

Figure 5DT‐13 downregulates PFKL by the c‐myc pathway. (a) mRNA expressions of eight genes related to PFKL modulation in HCC cells (*n* = 3,  ^∗^ *p* < 0.05 vs. DT‐13 [0 *μ*M]). (b) c‐myc expression in HCC cells. (c) Expression of c‐myc proteins in tumors. (d, e) Transfection was validated using RT‐PCR and western blot. (f) Effects of DT‐13 on the expression of c‐myc and PFKL in c‐myc‐EV and c‐myc‐OE HCC cells. (g) Effects of DT‐13 on the aerobic and glycolysis of c‐myc‐EV and c‐myc‐OE HCC cells (*n* = 3,  ^∗^ *p* < 0.05 vs. DT‐13 [0 *μ*M], ^#^ *p* < 0.05 vs. c‐myc‐EV + DT‐13 [0 *μ*M]). (h) Transfection was validated using western blot. (i) Effects of PFKL knockdown on the aerobic glycolysis of c‐myc‐OE HCC cells treated with DT‐13 (*n* = 3,  ^∗^ *p* < 0.05). (j) Effects of DT‐13 on the c‐myc and PFKL expression of sh‐c‐myc HCC cells.

**Figure:**
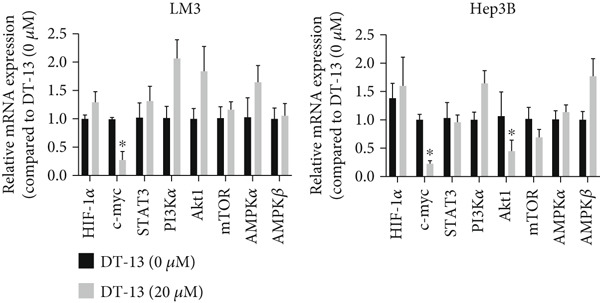
(a)

**Figure:**
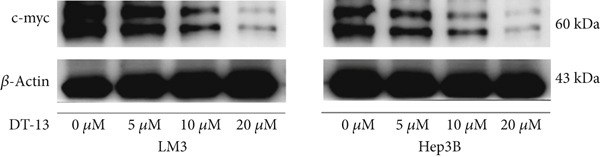
(b)

**Figure:**
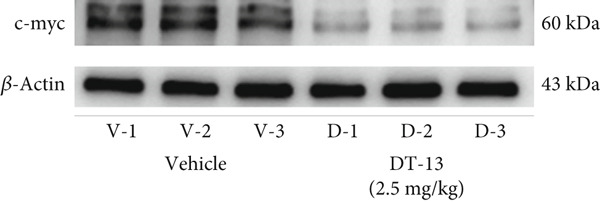
(c)

**Figure:**
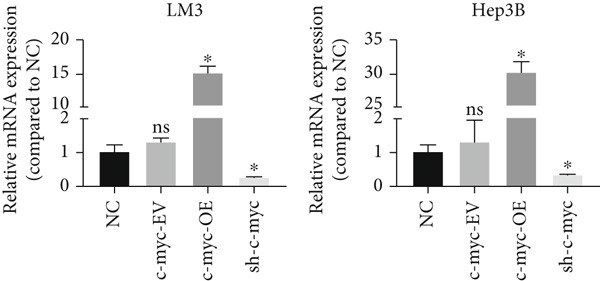
(d)

**Figure:**
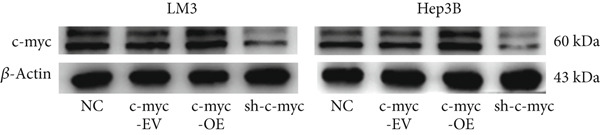
(e)

**Figure:**
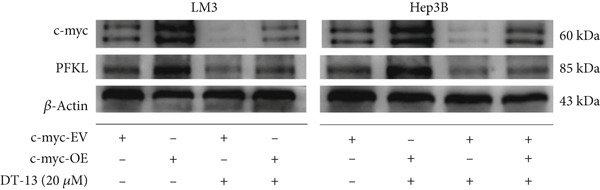
(f)

**Figure:**
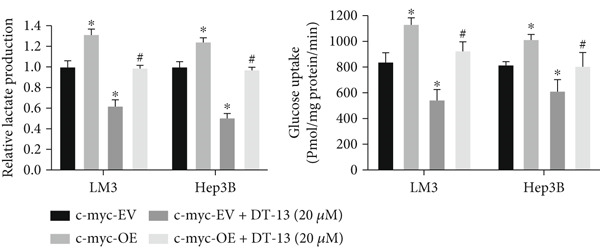
(g)

**Figure:**
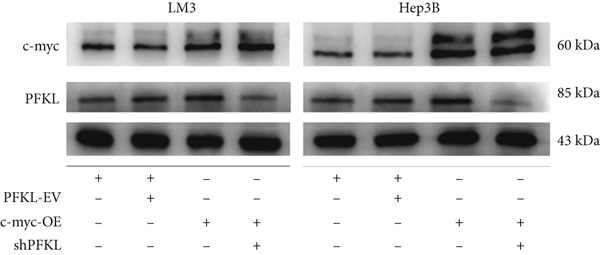
(h)

**Figure:**
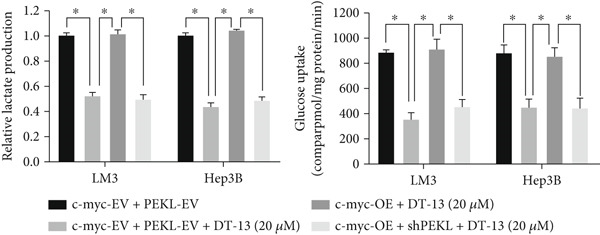
(i)

**Figure:**
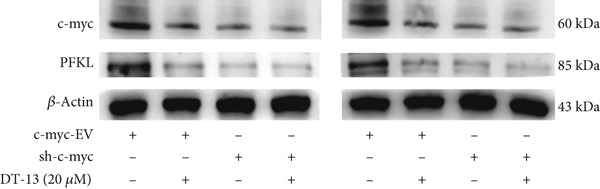
(j)

To determine whether PFKL suppression by DT‐13 was mediated via the c‐myc pathway, lentiviruses encoding c‐myc‐OE or knockdown (sh‐c‐myc) were introduced into HCC cells. Transduction efficiency was confirmed by PCR (Figure 5d) and western blotting (Figure 5e). In c‐myc‐EV cells, DT‐13 decreased both c‐myc and PFKL expressions; these inhibitory effects were reversed when c‐myc was overexpressed (Figure 5f). Similarly, DT‐13 reduced lactate production and glucose uptake in c‐myc‐EV cells, but these effects were reversed by c‐myc‐OE (Figure 5g).

Furthermore, c‐myc‐OE upregulated PFKL expression, leading to increased lactate production and glucose uptake, while subsequent knockdown of PFKL reversed these effects (Figure 5h,i). DT‐13 failed to reduce PFKL expression in sh‐c‐myc cells, indicating that c‐myc is required for DT‐13‐mediated suppression of PFKL (Figure 5j). Taken together, these findings demonstrate that DT‐13 downregulates PFKL expression and glycolytic activity in HCC cells via the c‐myc pathway.

### 3.6. DT‐13 Enhanced HCC′s Sensitivity to Sorafenib

The FDA approved sorafenib as the first‐line therapy for advanced HCC, and among its mechanisms, it is known to increase the aerobic glycolysis of HCC cells and inhibit OXPHOS [23]. Furthermore, it has been observed that suppressing HCC aerobic glycolysis by lentivirus or drugs could increase the efficiency of sorafenib on HCC [19, 24]. Here, it was observed that DT‐13 not only suppressed aerobic glycolysis but also increased the OXPHOS of HCC cells.

This study also investigated whether DT‐13 can enhance the chemosensitivity of sorafenib through a 48‐h treatment of HCC‐LM3 and Hep3B cells with sorafenib before detecting their viability. The IC_50_ values of LM3 and Hep3B were observed as 14.70 and 14.55 *μ*M, respectively (Figure 6a). The ratio of the IC_50_ of sorafenib to that of DT‐13 on the HCC cell lines was approximately 1:1. Furthermore, DT‐13 and sorafenib were combined at a 1:1 ratio to investigate their effects on aerobic glycolysis as well as HCC cells′ proliferation and apoptosis. The data indicated that compared to DT‐13 or sorafenib monotherapies, the cotreatment with DT‐13 and sorafenib resulted in increased cytotoxicity in HCC cells (Figure 6a). The results of median dose‐effect analysis by CalcuSyn software showed that the combination index (CI) of 10 *μ*M DT‐13 and 10 *μ*M sorafenib was < 1 in both HCC cells, indicating a synergistic effect of DT‐13 and sorafenib. Moreover, flow cytometry results revealed that 10 *μ*M sorafenib increased the HCC cell′s apoptosis rate, which was further enhanced by the addition of 10 *μ*M DT‐13 (Figure 6b). Since 10 *μ*M sorafenib significantly inhibited the colony formation of HCC cells, it was difficult to determine whether the inhibition of colony formation by sorafenib could be further strengthened (Figure 6c). Furthermore, sorafenib treatment increased lactate production, glucose consumption, and PFKL protein expression while reducing OXPHOS (Figure 6d,e). These modulatory effects on the above phenotypes by sorafenib could be reversed by DT‐13 treatment (Figure 6d,e).

Figure 6Results of in vitro and in vivo experiments showing HCC cells′ enhanced sensitivity to sorafenib after DT‐13 treatment. (a) Following a 48‐h treatment of HCC‐LM3 and Hep3B cells with DT‐13 and/or sorafenib, CI was calculated using CalsuSyn software. (b) Apoptosis rate following a 48‐h treatment of HCC‐LM3 and Hep3B cells with DT‐13 and/or sorafenib (*n* = 3,  ^∗^ *p* < 0.05 vs. vehicle, ^#^ *p* < 0.05 vs. Sora [10 *μ*M]). (c) The effects of DT‐13 and/or sorafenib on the colony formation of HCC cells. (d) The effects of DT‐13 and/or sorafenib on the lactate production and glucose uptake of HCC cells (*n* = 3,  ^∗^ *p* < 0.05 vs. vehicle, ^#^ *p* < 0.05 vs. Sora [10 *μ*M]). (e) Protein expression of PFKL, ATP5A1, and UQCRC2 in HCC cells after DT‐13 and/or sorafenib treatment for 48 h. (f) Effects of DT‐13 and/or sorafenib on the body weight of mice (*n* = 6, *p* > 0.05). (g) Effects of DT‐13 and/or sorafenib on the tumor volume of mice (*n* = 6,  ^∗^ *p* < 0.05). (h) Ki‐67 staining and TUNEL staining of tumor sections (magnification 200×).

**Figure:**
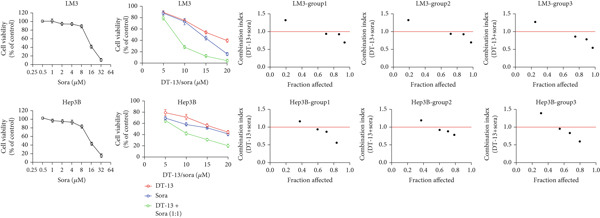
(a)

**Figure:**
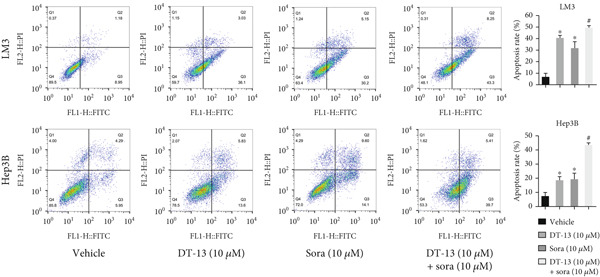
(b)

**Figure:**
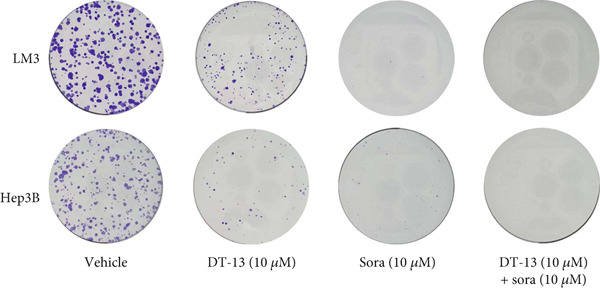
(c)

**Figure:**
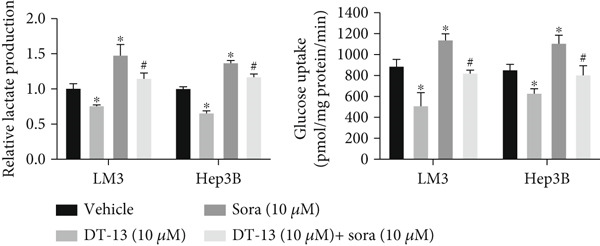
(d)

**Figure:**
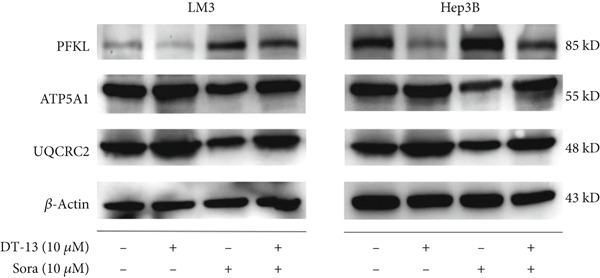
(e)

**Figure:**
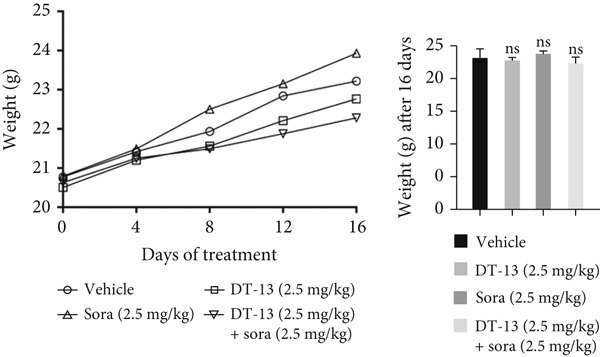
(f)

**Figure:**
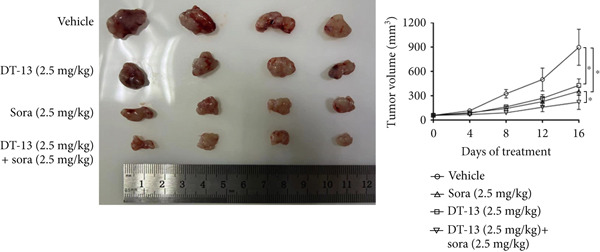
(g)

**Figure:**
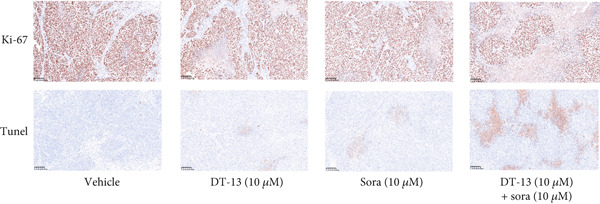
(h)

The potential of DT‐13 to increase the sensitivity of sorafenib against the Huh7‐SR cell line was investigated. The CCK‐8 assay showed that the IC_50_ of Huh7 to sorafenib was 3.19 *μ*M, and the IC_50_ of Huh7‐SR was 10.17 *μ*M (Figure S4A). The Huh7‐SR cell line was treated with DT‐13 and/or sorafenib in a 1:1 ratio. Compared with either DT‐13 or sorafenib used alone, the DT‐13–sorafenib combination showed significantly higher cytotoxicity against the Huh7‐SR cell line. CalcuSyn‐based median‐dose analysis revealed a CI < 1 for 10 *μ*M DT‐13 plus 10 *μ*M sorafenib in Huh7‐SR cells, confirming a synergistic interaction between the two agents (Figure S4B). The research further established a xenograft model with HCC‐LM3 cells to evaluate the potential of DT‐13 to increase the cells′ in vivo sensitivity to sorafenib. The 16‐day oral administration of 2.5 mg/kg DT‐13 and/or sorafenib did not influence the body weight of mice (Figure 6f). DT‐13 and sorafenib monotreatment significantly decreased the xenograft tumor volumes, which were further reduced after cotreatment (Figure 6g). The Ki‐67 and TUNEL staining validated that sorafenib suppressed the proliferation of HCC cells and induced apoptosis in vitro, with these effects further enhanced by cotreatment (Figure 6h).

In summary, the in vitro and in vivo findings suggest that DT‐13 boosted sorafenib′s effectiveness against HCC.

## 4. Discussion

Reprogrammed energy metabolism has been identified as a carcinomatous hallmark. Most tumor cells fulfill their anabolic needs by reprogramming cellular glucose metabolism, and HCC cells may exhibit the most comprehensive reprogramming of glucose metabolism among tumor cells [23]. This is achieved by inhibiting certain enzymes required for mature liver cell function but not for HCC cell function, as well as by stimulating enzymes that enhance glucose metabolism in the tumor cell. Therefore, this metabolic difference between HCC and normal liver cells can selectively target HCC cells [25]. Agents and lentiviruses that target key enzymes of aerobic glycolysis have been reported to exert therapeutic effects on HCC [6, 9, 19, 24]. For instance, knockdown of liver‐specific HK2 inhibits the development of diethyl nitrosamine (DEN)–induced liver cancer in mice. Moreover, silencing HK2 inhibits the growth and aerobic glycolysis in HCC cells while promoting OXPHOS and apoptosis [26]. Similarly, inhibiting HK2 increases HCC cells′ responsiveness to sorafenib and metformin. Moreover, no adverse physiological consequences were found in systemic HK2 knockout mice [26]. Therefore, it can be inferred that the application of drugs targeting aerobic glycolysis is a promising strategy against HCC.

DT‐13, obtained from the traditional Chinese medicine *Liriope muscari* (Decne) Baily, is among the drugs of research interest due to its various biological activities, including anti‐inflammatory, cardioprotective, hepatoprotective, immunomodulating, and antitumor effects [21]. It has been observed that DT‐13 has antitumor effects on various cancers, such as colorectal cancer [15], gastric cancer [14], prostate cancer [27], breast cancer [28], and acute myeloid leukemia [16]. Furthermore, it has been observed to inhibit the aerobic glycolysis of gastric and colorectal cancers [14, 15]. However, the anticancer effects of DT‐13 on HCC, as well as its mechanism of action, including a potential regulation of aerobic glycolysis, remain elusive. Here, in vitro experiments showed that DT‐13 dose‐dependently suppressed HCC cell proliferation and triggered apoptosis (Figure 1). Furthermore, in addition to its effectiveness, DT‐13 has become a research hotspot because of its safety. DT‐13, at a concentration of as low as 1.25 mg/kg, can suppress tumor growth in vivo [15]. In a mouse micronucleus assay, gavage intake of DT‐13 at doses up to 5000 mg/kg did not result in death, nor did it alter the general behavior or the gross appearance of the internal organs in mice. A subchronic toxicity assessment showed that 90‐day oral uptake at 10, 60, and 360 mg/kg produced no histopathological, biochemical, or hematological effects in Sprague–Dawley rats [22]. In the present study, DT‐13 did not affect the apoptosis rate and cell viability of normal liver THLE‐2 cell lines in vitro (Figures 1 and 2). However, a gavage of 2.5 mg/kg DT‐13 significantly reduced the tumor volume but did not affect the mice′s body weight. The TUNEL and Ki‐67 staining analyses indicated that 2.5 mg/kg DT‐13 induced apoptosis in HCC cells and inhibited their proliferation in vivo (Figure 1). Moreover, 2.5 mg/kg DT‐13 had no effects on the serum levels of creatinine and ALT. The H&E staining of mice′s hearts, liver, lungs, and kidneys showed that a 16‐day oral intake of 2.5 mg/kg did not cause pathological damage in these organs (Figure 2). The efficacy and safety of DT‐13 in treating HCC have laid a solid theoretical foundation for its clinical application.

DT‐13 dose‐dependently suppressed glucose uptake, lactate production, and ATP production and increased OXPHOS in HCC cell lines. Meanwhile, DT‐13 reduced the NADPH levels of HCC cells, which may be related to the decreased glycolytic flux, resulting in a lower total amount of glucose‐6‐phosphate (G6P) entering the pentose phosphate pathway (PPP), leading to a decrease in NADPH production by the PPP [29, 30]. The underlying mechanism remains to be experimentally verified. Gavage of DT‐13 decreased the lactate level in the tumor tissues. Among the seven aerobic glycolysis–related genes, DT‐13 significantly reduced the mRNA and protein expression of PFFL in both HCC cell lines in vivo and in vitro (Figure 3). The findings indicated that DT‐13 suppressed the aerobic glycolysis of HCC cells. Moreover, DT‐13 did not affect the glycolysis of normal liver THLE‐2 cell lines (Figure 2).

PFK1 is the second rate‐limiting enzyme of aerobic glycolysis, which uses ATP to catalyze F‐6‐P to F‐1,6‐BP [19]. In human carcinomas, PFK1 is markedly activated and expressed to support aerobic glycolysis and proliferation [31]. Furthermore, it has been reported to modulate apoptosis by interaction with the proapoptotic protein Bad [12]. In HCC, PFK1 is primarily expressed as PFKL. Zhang et al. reported that a small molecular compound, dimethylaminomicheliolide (DMAMCL), exerted antitumor effects on neuroblastoma by inhibiting aerobic glycolysis and targeting PFKL. Furthermore, it induced neuroblastoma cell apoptosis, which was reversed after PFKL‐OE. Moreover, PFKL downregulation by siRNA transfection induced apoptosis of neuroblastoma cells [32]. Similarly, Zheng et al. indicated that Iodine‐125 (^125^I) inhibited aerobic glycolysis and malignant biological behavior of HCC cell lines via the miR338/PFKL pathway. This apoptosis induction by ^125^I on HCC cells might be attenuated by PFKL overexpression [33]. As stated above, DT‐13 significantly suppressed the mRNA of PFKL, HCC proliferation, and aerobic glycolysis, whereas it induced apoptosis. However, these effects were reversed after PFKL‐OE (Figure 4). PFKL knockdown induced HCC cell apoptosis as well as suppressed proliferation and aerobic glycolysis. However, DT‐13 treatment failed to modulate these phenotypes in sh‐PFKL HCC cells (Figure 4). These findings confirm that DT‐13 regulates HCC′s aerobic glycolysis, proliferation, and apoptosis by targeting the PFKL enzyme.

PFKL coordinates diverse aspects of tumor metabolism, including glycolysis, lipid turnover, and functional integration of mitochondria, offering broad opportunities for therapeutic intervention. In HCC cells exposed to energy stress, PFKL undergoes phosphorylation and interacts with Perilipin 2 (PLIN2) to promote lipid droplet–mitochondria tethering, which facilitates lipolysis and *β*‐oxidation, supporting tumor proliferation and survival [34]. Thus, inhibition of PFKL disrupts both glycolytic flux and lipid metabolism, more effectively inhibiting the tumor bioenergetics. Despite this potential, direct comparative studies evaluating the relative efficacy and adverse profiles of targeting distinct glycolytic enzymes in the same tumor model remain lacking. Work by Xu et al. [35] has highlighted isozyme‐specific regulation, for instance, HK2 downregulation versus PFKL upregulation in prostate cancer, but has not functionally compared the inhibition of these enzymes. Future investigations should therefore directly assess the antitumor efficacy and potential on‐target/off‐tumor toxicities of different glycolytic enzyme inhibitors within identical experimental settings. Such analyses are essential for rationally selecting the most effective and least toxic strategies for targeting tumor metabolism.

c‐myc is one of the widely studied oncogenes, regulating multiple malignant phenotypes, including cell cycle progression, apoptosis, proliferation, and remodeling of the tumor microenvironment [9, 36]. Regarding aerobic glycolysis, c‐myc has been reported to promote glycolysis flux by upregulating the expression of GLUT1, HK2, PFKL, PKM2, and LDHA [9, 37]. In this study, DT‐13 significantly inhibited the mRNA expression of c‐myc in both HCC cell lines. DT‐13 reduced the protein expression of c‐myc in both in vivo and in vitro studies. The inhibition of DT‐13 on the PFKL expression and aerobic glycolysis in HCC was reversed by c‐myc‐OE. Moreover, these effects were abolished upon PFKL depletion. Furthermore, in sh‐c‐myc HCC cells, DT‐13 was unable to reduce the expression of PFKL (Figure 5). Moreover, c‐myc has been reported to regulate glutaminolysis in cancer cells by simultaneously increasing glutamine uptake and its subsequent catabolism, which serves as another major source of NADPH [29, 38]. The inhibition of DT‐13 on the NADPH level in HCC may be partly due to the repression of c‐myc‐driven glutamine metabolism, which requires further experimental validation. These results suggest that DT‐13 inhibited the expression of PFKL via the c‐myc pathway, attenuating aerobic glycolysis in HCC. Although sorafenib is used as a first‐line treatment for advanced HCC, its high costs and resistance have limited its benefits to only a few patients [39]. Therefore, new chemotherapeutic options are urgently required to improve HCC′s sensitivity to sorafenib. Multiple mechanisms, including enhanced aerobic glycolysis, are involved in HCC′s resistance to sorafenib. In our previous work, it has been indicated that agents targeting aerobic glycolysis enzymes, such as aspirin (targeting PFKFB3) [24], sodium butyrate (targeting HK2) [6], proanthocyanidin B2 (targeting PKM2) [20], and EGCG (targeting PFKL) [13], could synergistically enhance the therapeutic effect of sorafenib on HCC. Here, in vitro and in vivo experiments showed that DT‐13 could enhance sorafenib′s antitumor effects by targeting PFKL (Figure 6). DT‐13 could exert a synergistic effect with sorafenib on the Huh7‐SR cells (Figure S4), highlighting its translational potential as an adjuvant to improve therapeutic responses in HCC.

## 5. Conclusion

**Figure 7:**
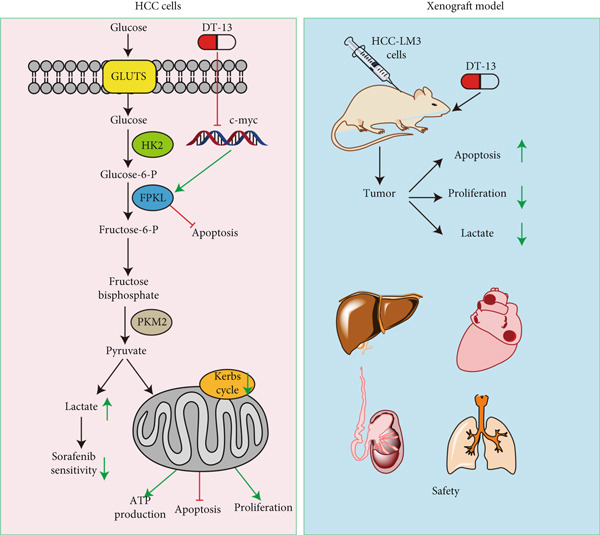
DT‐13 exerts anticancer effects on HCC cells in vivo and in vitro.

Overall, through in vitro and in vivo experiments, the current work revealed that DT‐13, a steroidal saponin derived from the tuber of *Liriope muscari* (Decne) Baily, inhibits the aerobic glycolysis, proliferation, and survival of HCC cells via the c‐myc/PFKL signaling axis. Moreover, DT‐13 was nontoxic to mice and normal liver THLE‐2 cells. Moreover, it inhibited the sorafenib‐induced increase of aerobic glycolysis in HCC cells, thereby enhancing the drug′s efficacy against HCC cells (Figure 7).

## Ethics Statement

The current study was approved by the Animal Care and Use Committee of Sir Run Run Shaw Hospital, Zhejiang University School of Medicine (No. SRRSH202102210).

## Consent

The authors have nothing to report.

## Conflicts of Interest

The authors declare no conflicts of interest.

## Author Contributions

Qiang Yu: conception, investigation, and writing. Liangning Hu: funding acquisition, methodology, and review. Chenfei Tan: funding acquisition and formal analysis. Min Gao: validation and supervision. Zhenzhen Wen: resources and project administration. Qiang Yu and Liangning Hu contributed equally to this work and share first authorship.

## Funding

This study was funded by the National Natural Science Foundation of China, 10.13039/501100001809, No. 82100202, and the Hangzhou Medical Health Science and Technology Project, A20230864.

## Supporting Information

Additional supporting information can be found online in the Supporting Information section.

## Supporting information


**Supporting Information 1** Primary antibodies used in western blot are listed in Table S1. PCR primers used in RT‐PCR are listed in Table S2.

**Supporting Information 2** Figure S1: DT‐13 elevated extracellular pH and reduced intracellular ATP and NADPH in HCC cells, indicating acute suppression of glycolysis and PPP flux. Figure S2: In silico docking placed DT‐13 into a surface pocket of PFKL. Figure S3: DT‐13 selectively downregulated PFKL mRNA and protein without affecting PFKM or PFKP isoforms, confirming isoform‐specific transcriptional suppression. Figure S4: DT‐13 lowered the sorafenib IC_50_ of Huh7‐SR cells (*μ*M) and yielded a combination index (CI) < 1, indicating strong synergistic resensitisation to sorafenib in vitro.

## Data Availability

The data that support the findings of this study are available from the corresponding author upon reasonable request.
