# Supplementary material for: PFKL Inhibition by DT‐13: A Novel Approach to Combat Hepatocellular Carcinoma
Source: Int J Hepatol. 2025 Dec 12;2025:5211859. doi: 10.1155/ijh/5211859 (PMC12752837; doi:10.1155/ijh/5211859)
Supplement: Supplementary file 1 — Supporting Information 1 Primary antibodies used in western blot are listed in Table S1. PCR primers used in RT‐PCR are listed in Table S2. [file IJH-2025-5211859-s002.docx]

**Supplementary Table 1** The primary antibodies used in the study

| Antibody | Species | Dilution ratio | Supplier | Catalogue number |
| --- | --- | --- | --- | --- |
| β-actin | M | 1:1000 | CST | 3700 |
| PCNA | Rbt | 1:5000 | Proteintech | 10205-2-AP |
| Bcl-2 | Rbt | 1:1000 | Proteintech | 26593-1-AP |
| PFKL | M | 1:5000 | Proteintech | 68385-1-Ig |
| UQCRC2 | Rbt | 1:2000 | Proteintech | 14742-1-AP |
| ATP5A1 | Rbt | 1:5000 | Proteintech | 14676-1-AP |
| c-myc | M | 1:5000 | Proteintech | 67447-1-Ig |
| PFKP | M | 1:5000 | Proteintech | 68129-1-Ig |
| PFKM | M | 1:5000 | Proteintech | 55028-1-AP |

Abbreviations for the table: M mouse; Rbt rabbit; CST Cell Signaling Technology (Danvers, MA, USA).

**Supplementary Table 2** Primers used for rt-PCR

| Gene name | Forward (5′-3′) | Reverse (5′-3′) |
| --- | --- | --- |
| β-actin | CTGGAACGGTGAAGGTGACA | AAGGGACTTCCTGTAACAATGCA |
| GLUT1 | GCCAGAAGGAGTCAGGTTCAA | TCCTCGGAAAGGAGTTAGATCC |
| HK2 | GAGCCACCACTCACCCTACT | CCAGGCATTCGGCAATGTG |
| PFKFB3 | TTGGCGTCCCCACAAAAGT | AGTTGTAGGAGCTGTACTGCTT |
| PFKL | GTACCTGGCGCTGGTATCTG | CCTCTCACACATGAAGTTCTCC |
| PKM2 | ATGTCGAAGCCCCATAGTGAA | TGGGTGGTGAATCAATGTCCA |
| LDH-A | ATGGCAACTCTAAAGGATCAGC | CCAACCCCAACAACTGTAATCT |
| LDH-B | TGGTATGGCGTGTGCTATCAG | TTGGCGGTCACAGAATAATCTTT |
| HIF-1α | GAACGTCGAAAAGAAAAGTCTCG | CCTTATCAAGATGCGAACTCACA |
| c-myc | GTCAAGAGGCGAACACACAAC | TTGGACGGACAGGATGTATGC |
| AMPK-α | TTGAAACCTGAAAATGTCCTGCT | GGTGAGCCACAACTTGTTCTT |
| AMPK-β | CCACTCCGAGGAAATCAAGGC | CTGGGCGGGAGCTTTATCA |
| STAT3 | CAGCAGCTTGACACACGGTA | AAACACCAAAGTGGCATGTGA |
| PI3K | TATTTGGACTTTGCGACAAGACT | TCGAACGTACTGGTCTGGATAG |
| Akt1 | AGCGACGTGGCTATTGTGAAG | GCCATCATTCTTGAGGAGGAAGT |
| mTOR | GCAGATTTGCCAACTATCTTCGG | CAGCGGTAAAAGTGTCCCCTG |
| PFKP | GCATGGGTATCTACGTGGGG | CTCTGCGATGTTTGAGCCTC |
| PFKM | AGCGTTTCGATGATGCTTCAG | GGAGTCGTCCTTCTCGTTCC |
