# Supplementary material for: PFKL Inhibition by DT‐13: A Novel Approach to Combat Hepatocellular Carcinoma
Source: Int J Hepatol. 2025 Dec 12;2025:5211859. doi: 10.1155/ijh/5211859 (PMC12752837; doi:10.1155/ijh/5211859)
Supplement: Supplementary file 2 — Supporting Information 2 Figure S1: DT‐13 elevated extracellular pH and reduced intracellular ATP and NADPH in HCC cells, indicating acute suppression of glycolysis and PPP flux. Figure S2: In silico docking placed DT‐13 into a surface pocket of PFKL. Figure S3: DT‐13 selectively downregulated PFKL mRNA and protein without affecting PFKM or PFKP isoforms, confirming isoform‐specific transcriptional suppression. Figure S4: DT‐13 lowered the sorafenib IC50 of Huh7‐SR cells (μM) and yielded a combination index (CI) < 1, indicating strong synergistic resensitisation to sorafenib in vitro. [file IJH-2025-5211859-s001.docx]

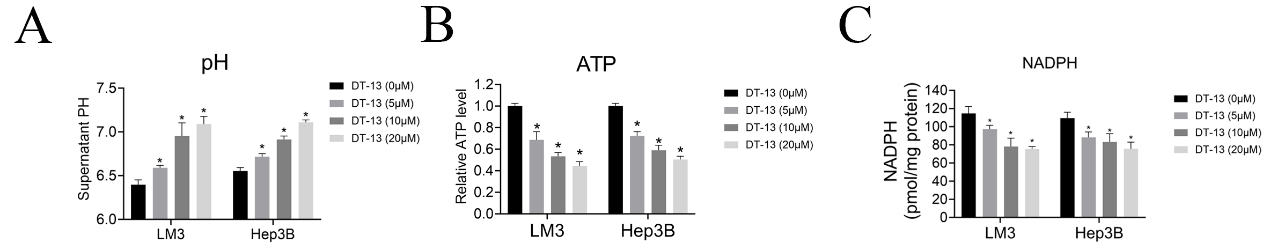


Figure S1. Effects of DT-13 on PH, ATP production and NADPH levels.(A) PH levels of supernatant (n=3, *P < 0.05 vs DT-13 (0 μM)). (B) Effects of DT-13 on the ATP production (n=3, *P < 0.05 vs DT-13 (0 μM)). (C) Effects of DT-13 on the NADPH levels (n=3, *P < 0.05 vs DT-13 (0 μM)).


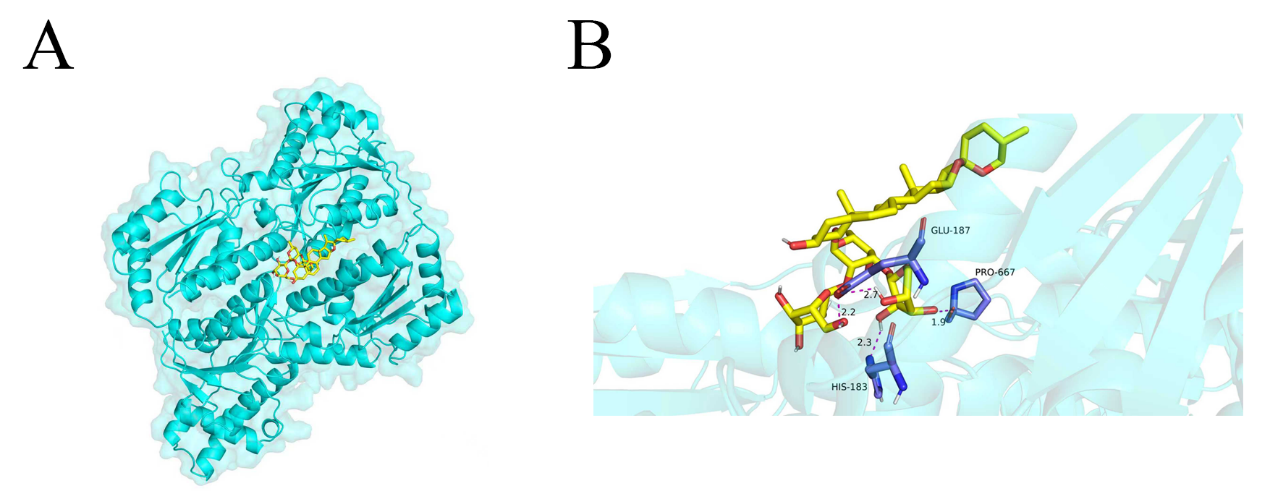


Figure S2. Docked structure and interactions of DT-13 binding to PFKL. (A) DT-13 binds to a cavity on the surface of the PFKL protein. (B) Detailed interactions (formation of 4 hydrogen bonds) between DT-13 and amino acid residues of PFKL.


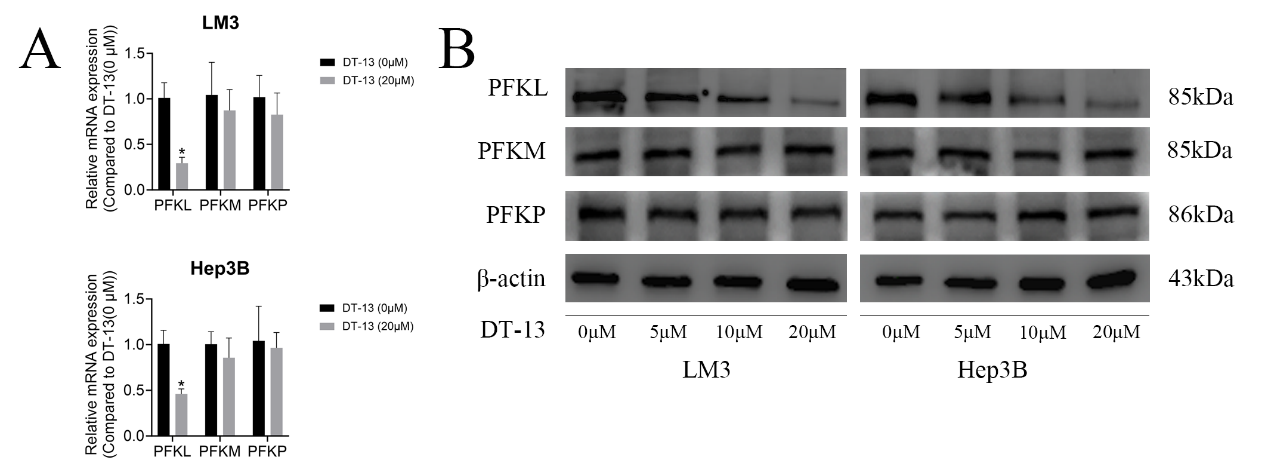


Figure S3. Effects of DT-13 on the expression of PFKL, PFKM, and PFKP. (A) mRNA expressions of PFKL, PFKM, and PFKP in HCC cells (n=3, *P < 0.05 vs DT-13 (0 μM)). (B) Protein expression of PFKL, PFKM, and PFKP detected by western blot.


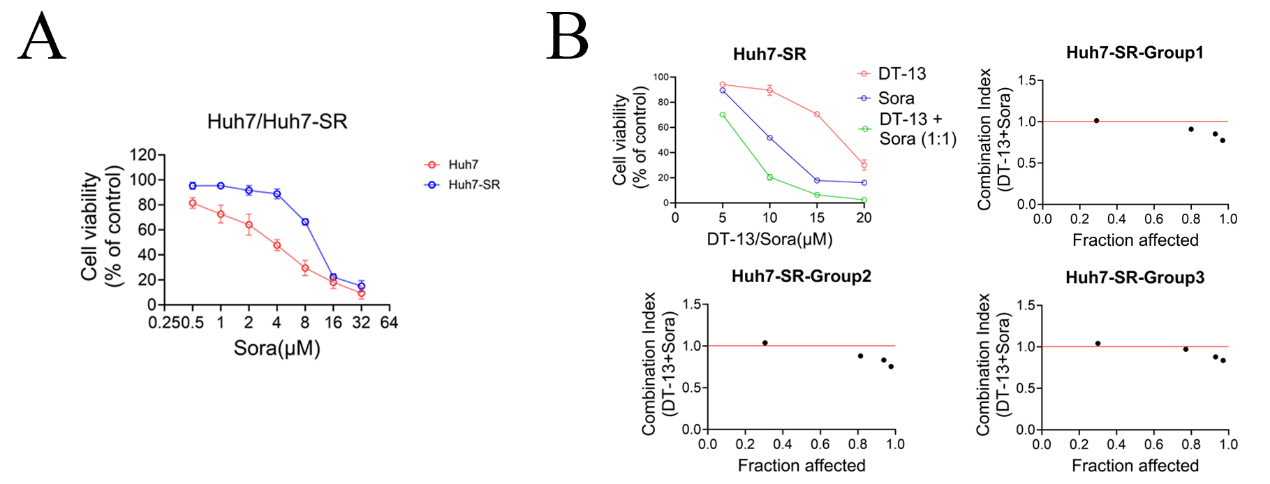


Figure S4. DT-13enhanced sensitivity of Huh-SR to sorafenib *in vitro*. (A) IC50 of Huh7 and Huh7-SR cells to sorafenib calculated by CCK-8. (A) Following a 48-h treatment of Huh7-SR cells with DT-13 or/and sorafenib, CI was calculated using CalsuSyn software.
